# The effectiveness of virtual reality for rehabilitation of Parkinson disease: an overview of systematic reviews with meta-analyses

**DOI:** 10.1186/s13643-022-01924-5

**Published:** 2022-03-19

**Authors:** Yaqin Lu, Yonggui Ge, Wanqiang Chen, Wenting Xing, Lushan Wei, Caixia Zhang, Yusheng Yang

**Affiliations:** Department of Rehabilitation, The First Hospital of Lanzhou University, No.1 Donggang West Road, Chengguan District, Lanzhou, 730000 People’s Republic of China

**Keywords:** Virtual reality, Parkinson disease, Effectiveness, Overview, Systematic reviews, Meta-analyses

## Abstract

**Background:**

An increasing number of systematic reviews (SRs) and meta-analyses (MAs) of clinical trials have begun to investigate the effects of virtual reality (VR) in patients with Parkinson disease (PD). The aim of this overview was to systematically summarize the current best evidence for the effectiveness of VR therapy for the rehabilitation of people with PD.

**Methods:**

We searched SR-MAs based on randomized controlled trials (RCTs) for relevant literature in PubMed, Embase, and Cochrane library databases for systematic reviews from inception to December 5, 2020, and updated to January 26, 2022. The methodological quality of included SR-MAs was evaluated with the Assessing the Methodological Quality of Systematic Reviews 2 (AMSTAR-2), and the certainty of evidence for outcomes with the Grading of Recommendations, Assessment, Development and Evaluation (GRADE). We created an evidence map using a bubble plot format to represent the evidence base in 5 dimensions: effect size of VR therapy versus active intervention (AT), clinical outcome area, number of trials, statistical significance, and certainty of evidence.

**Results:**

From a total of 585 reports, 12 reviews were identified, of which only one was rated moderate quality, three were rated low quality, and eight were rated critically low quality by AMSTAR 2. Compared with AT, VR therapy induced increased benefits on stride/step length, balance, and neuropsychiatric symptoms. Compared with passive intervention (PT), VR therapy had greater effects on gait speed, stride/step length, balance, activities of daily living, and postural control in people with PD. Certainty of evidence varied from very low to moderate.

**Conclusions:**

We found the methodological quality of the reviews was poor, and certainty of the most evidence within them was low to very low. We were therefore unable to conclude with any confidence that, in people with PD, VR therapy is harmful or beneficial for gait, balance, motor function, quality of life, activities of daily living, cognitive function, neuropsychiatric symptoms, and postural control. In the future, rigorous-designed, high-quality RCTs with larger sample sizes are needed to further verify the effectiveness of VR therapy in the treatment of PD.

**Supplementary Information:**

The online version contains supplementary material available at 10.1186/s13643-022-01924-5.

## Background

Parkinson disease (PD) is the most common progressive neurodegenerative disease worldwide. PD prevalence is increasing with age and affects 1% of the population above 60 years [1]. It is estimated that by around 2030, the number of PD patients in China will reach 5 million, accounting for about 50% of the total number of PD patients in the world [2]. PD is characterized by motor symptoms such as rest tremor, bradykinesia, rigidity, and postural instability, which affect gait, balance, and movement quality, leading to difficulty in performing basic daily activities and quality of life and placing a heavy burden on families and society [3]. Multidisciplinary input is increasingly recognized as important in PD management [4]. Currently, drugs and surgical approaches were the main treatments of PD. Clinically approved drug treatments for PD mainly include levodopa, dopaminergic receptor agonists, and monoamine oxidase-B inhibitors. Levodopa is considered as a “first line” drug, but the long-term use of it leads to many complications [5]. Deep brain stimulation may be an effective treatment in PD patients; however, clinical trials have shown that it may have cognitive and psychiatric side effects [6]. Conventional rehabilitation is considered as an adjuvant to pharmacological and surgical treatments for PD to improve many dysfunctions and self-care ability, even delay the progression of the disease.

Virtual reality (VR) has emerged as a promising technology for researching complex impairments in people with PD and for providing personalized rehabilitation [7]. This technology typically combines real-time motion detection within a virtual environment in the context of a (video)game. The user can perceive, feel, and interact with virtual environments, viewing an avatar (a character or graphical representation of the user) that mimics the user’s movements [8] by multiple sensory channels such as sight, sound, and touch [9]. Immediate feedback about performance and success is provided both concurrently (during game play) and terminally (at the end of the game). VR therapy attempts to promote activity-dependent neuroplasticity and motor learning [10, 11]. Recently, numerous systematic reviews (SRs) and meta-analyses (MAs) based on randomized controlled trials (RCTs) regarding the clinical effectiveness of VR therapy in the treatment of PD have been published. However, the overall results have remained mixed or inconclusive and their quality is uneven. An overview of SR-MAs is a relatively new method that aims to support clinical decision-making by synthesizing the findings, critically appraising the quality, and attempting to resolve discordant outcomes.

Therefore, we conducted an overview of SR-MAs to identify and summarize the existing evidence and to systematically determine the clinical effectiveness of using VR therapy to treat PD.

## Methods

The overview was completed according to the Preferred Reporting Items for Systematic Reviews and Meta-Analyses (PRISMA) [12] and the guidelines recommended by the Cochrane Collaboration [13]. The PRISMA checklist can be found in Additional file 1. The protocol was not prospectively registered.

### Search strategy

We systematically searched PubMed, Embase, and Cochrane library databases for systematic reviews from inception to December 5, 2020, and updated to January 26, 2022. We used a combination of Medical Subject Headings with Entry Terms, or EMTREE with keywords as follows: *Parkinson Disease*, *Virtual Reality Exposure Therapy*, *Virtual Reality*, *Exergaming*, *Systematic Review*, and *Meta-Analysis.* In addition, to ensure a comprehensive data collection, references of relevant reviews were searched manually to identify additional eligible studies. The search strategy for the PubMed database is shown in Additional file 2.

### Eligibility criteria

#### Types of reviews

In this overview, we have included SR-MAs of RCTs, and the full-text article was published in the English language. A review qualified as a SR-MA if, at a minimum, it had been conducted with systematic methods, an attempt was made to identify all of the relevant primary studies in at least one database and a search strategy was provided, and it performed a quality appraisal of the primary trials included and included quantitative syntheses. The reason for this is the fact that meta-analytical studies offer an effect estimate which would facilitate data analysis, but this was not the case for systematic reviews.

#### Types of participants

Participants involved in reviews were clinically definite diagnosis of PD and were defined by the UK Parkinson’s Disease Society Brain Bank or other diagnostic criteria. We had no restrictions on gender, age, drug dosage, duration, and severity of PD. We included reviews reporting an intervention carried out in a mixed sample of participants if data for participants with PD were provided separately.

#### Types of interventions

Intervention groups were VR-based rehabilitation interventions (with/without combined interventions). Control interventions needed to involve passive treatment (PT) or active treatment (AT) without a VR component. PT included either educational programs or a control group receiving no intervention. AT involved usual care or any other exercise intervention without a VR component.

#### Types of outcome measures

The primary outcomes we collected included two aspects: (1) Gait. Gait speed, stride/step length, walking stability such as the Dynamic Gait Index (DGI) or Functional Gait Assessment (FGA), and walking distance such as the Two- or Six-Minute Walk Test (2MWT or 6MWT) were used to evaluate gait. (2) Balance function. Balance was assessed with Berg Balance Scale (BBS), Timed Up and Go test (TUG), Single-Leg Stance Test (SLS), or Mini-Balance Evaluation Systems Test (Mini-BESTest).

The secondary outcomes included the following: (1) Balance confidence. The Falls Efficacy Scale (FES), FES-international (FES-I), and Activities-specific Balance Confidence scale (ABC) were used to measure the patient’s level of confidence in doing specific activities that could affect balance and cause falls. (2) Motor function. We used the Unified Parkinson’s Disease Rating Scale (UPDRS) part III to address global motor function changes. (3) Quality of life. Quality of life was determined by the 39-Item Parkinson’s Disease Questionnaire (PDQ-39), or its short form (PDQ-8), or the World Health Organization Quality of Life for Older Persons (WHOQOL-OLD). (4) Activities of daily living. UPDRS part II and the modified Barthel Index (MBI) were employed to measure activities of daily living. (5) Cognitive function. Cognitive function was measured by Montreal Cognitive Assessment (MoCA), Digit Span forward (DSF), and Mini-Mental State Examination (MMSE). (6) Neuropsychiatric symptoms. Beck Anxiety Inventory (BAI), Beck Depression Inventory (BDI), Hamilton Depression Scale (HAMD), Hospital Anxiety and Depression Scale (HADS), and 15-item Geriatric Depression Scale (GDS-15) were used to record neuropsychiatric symptom changes in subjects. (7) Postural control. Sensory organization test (SOT) was designed to examine the degree of postural control.

The exclusion criteria included the following: (1) studies which had mixed samples (PD, stroke, multiple sclerosis, cerebral palsy, or other neurological disorders) cannot extract data separately; (2) studies where PD patients all used VR without control group or control group was healthy individuals; (3) studies where PD patients with different symptoms (freezers vs. non- freezers) underwent the same VR therapy; and (4) non-systematic reviews, guidelines, conference abstracts, surveys, commentaries, editorials, letters, and notes.

### Study selection

All titles and abstracts were initially screened by two independent investigators (L.Y.Q and G.Y.G) after automatically removing duplicate results to identify potentially relevant studies for inclusion. At this stage, we excluded studies that were not focused on the effects of VR therapy on PD patients or not described as SR-MAs. Furthermore, full-text articles were reviewed and selected according to eligibility criteria. We excluded reviews that did not present summary statistics for outcomes (effect size with 95% CIs). Final relevant studies were shortlisted. In case of discrepancies, a consensus was achieved by discussion. If consensus could not be reached, a third reviewer (Y.Y.S) was consulted.

### Data extraction

Two investigators (L.Y.Q and G.Y.G) extracted the following basic characteristics from each eligible review: the first author, publication year, country of the review author, the number of included studies, sample size, interventions (experiment interventions and control interventions), outcomes of interest, quality assessment tools, and main conclusions. Differences between the review authors were settled by discussion, and a third reviewer (Y.Y.S) was consulted if differences persisted. The study authors were contacted with the aim of acquiring additional information on the data presented.

### Quality assessment

Two independent investigators (L.Y.Q and G.Y.G) assessed the methodological quality of the SR-MAs and the certainty of evidence in the included SR-MA. We resolved discrepancies through discussion or, if needed, through arbitration by a third review author (Y.Y.S).

#### Methodological quality of included SR-MAs

The methodological quality of each included review was evaluated using the Assessing the Methodological Quality of Systematic Reviews 2 (AMSTAR-2) tool [14]. AMSTAR-2 is a comprehensive critical appraisal tool for SRs/MAs of randomized and non-randomized studies that focuses on weaknesses in critical domains but not an overall score. The tool assesses 16 items, among which 7 are critical domains (items 2, 4, 7, 9, 11, 13, and 15). The evaluation is reduced to three options, “Yes,” “Partial Yes,” and “No.” AMSTAR-2 classifies the overall confidence on the results of the review into four levels: high, moderate, low, and critically low.

#### Certainty of evidence in included SR-MAs

We did not re-evaluate the certainty of the evidence for the main outcomes if the review author had already performed the assessment. We used the Grading of Recommendations Assessment, Development and Evaluation (GRADE) assessment from the pooled outcome data as assessed by authors in a particular systematic review. Where review authors did not undertake GRADE, we performed a new assessment ourselves. The GRADE scoring is judged by the risk of bias, inconsistency, imprecision, indirectness, and publication bias [15]. Results are divided into four levels: high, moderate, low, and very low.

### Statistical analysis

We did not conduct novel analyses for this overview. We summarized the characteristics of included reviews as well as the AMSTAR-2 ratings for each separate review. We have presented comparisons for each primary and secondary outcome where possible. Comparisons of primary interest were as follows.

- VR therapy versus AT
- VR therapy versus PT
- VR therapy versus controls (mixed AT with PT)

We created a bubble plot to present evidence base using Microsoft office Excel 2016 software (Microsoft Corp, Redmond, WA, www.microsoft.com). Each bubble plot displayed information in 5 dimensions: effect size (standard mean difference (SMD) or mean difference (MD)) of VR therapy for PD patients (*y*-axis), clinical outcome area (*x*-axis), number of trials (bubble size), statistical significance (bubble pattern), and certainty of evidence (bubble color).

## Results

### Search results

**Fig. 1:**
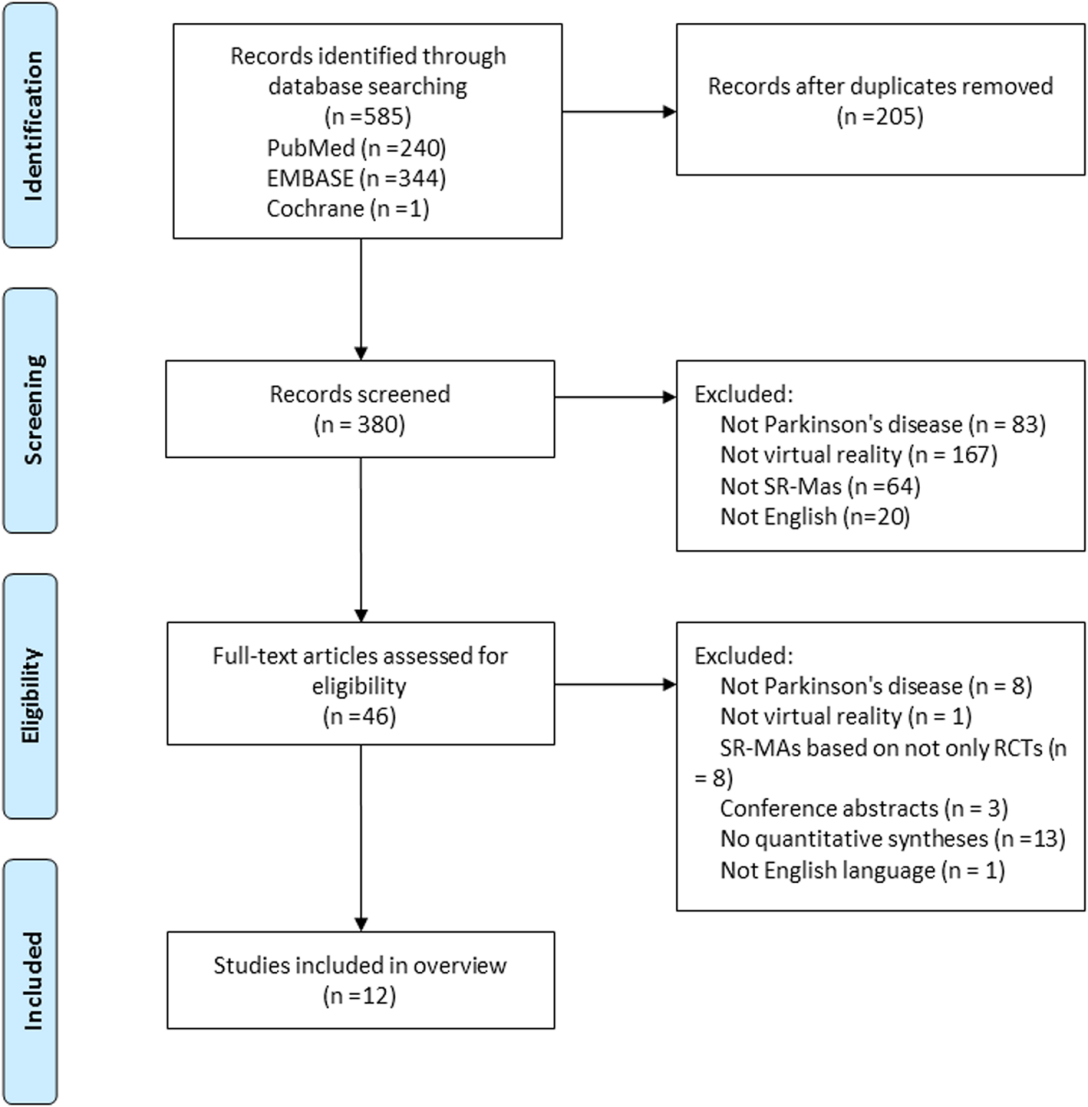
A flow diagram of study screening and selection procedures

A flow diagram of study screening and selection procedures is illustrated in Fig. 1. Our electronic search yielded 585 potentially relevant publications. After automatic removal of duplicates, 380 records were screened on the basis of the title or abstract. Of the remaining 46 reviews, 34 reviews were excluded: participants were not PD (*n* = 8), intervention was not VR (*n* = 1), SR-MAs were not based on RCTs (*n* = 8), conference abstracts only (*n* = 3), systematic review without quantitative data syntheses (*n* = 13), and full text was not English language (*n* = 1). Finally, 12 SR-MAs [16–27] met the inclusion criteria and were included in this overview.

### Study characteristics

The characteristics of the 12 SR-MAs included in our final analysis are summarized in Table 1. All studies were published between 2015 and 2021. The number of apposite studies included in each review ranged from 2 to 22, and the sample sizes ranged from 74 to 901. All reviews reported the VR-based rehabilitation training (VR therapy) as interventions. Out of the eligible SR-MAs, seven [16, 18, 21–25] included VR therapy versus AT as a comparison, two [19, 26] included VR therapy versus AT or PT respectively as comparisons. Two reviews [17, 20] did not classify the control group, which mixed AT with PT. In addition, one review [27] presented two evidence syntheses that were derived from single studies respectively. Six SR-MAs [16, 19–21, 25, 26] used the Cochrane Collaboration’s tool, and six SR-MAs [17, 18, 22–24, 27] used the PEDro scale.

**Table 1:** Characteristics of the included systematic reviews

Author (year)	Country	Trials (sample size)	Intervention		Outcomes	Quality assessment tool	Main conclusions
			Experiment	Control			
Sarasso E (2021) [16]	Italy	22 (901)	VR	AT	Gait speed Stride/step length Walking stability (DGI/FGA) Walking distance (6MWT) Balance (BBS) Balance (TUG) Balance confidence (ABC/FES/FES-I) Motor function (UPDRS-III) Quality of life (PDQ-39/PDQ-8)	Cochrane Collaboration’s tool	Balance training in a VR setting is more effective than balance training to improve balance in PD subjects immediately after training.
Li R (2021) [17]	China	22 (836)	VR	Both (mix AT with PT)	Balance (BBS) Quality of life (PDQ-39) Activities of daily living (MBI/UPDRS-II) Neuropsychiatric symptoms (HAMD/HADS/GDS-15/BDI)	PEDro scale	Rehabilitation training based on VR significantly improved balance, quality of life, activities of daily living, and depressive symptoms compared to the control group.
Elena P (2021) [18]	Cyprus	14 (548)	VR	AT	Walking stability (DGI) Balance (BBS) Balance (TUG) Balance confidence (ABC) Quality of life (PDQ-39) Activities of daily living (UPDRS-II)	PEDro scale	VR therapy related with improved quality of life, balance, and gait.
Triegaardt J (2020) [19]	UK	10 (343)	VR	AT; PT	Gait speed Stride/step length Balance (BBS) Motor function (UPDRS-III) Cognitive function (MoCA) Activities of daily living (UPDRS-II) Quality of life (PDQ39/PDQ8)	Cochrane Collaboration’s tool	Compared with AT, VR therapy led to greater improvement of stride length. Compared with PT, VR therapy had greater effects on gait speed, stride length, balance, and activities of daily living.
Marotta N (2020) [20]	Italy	7 (236)	VR	Both (mix AT with PT)	Functional locomotion	Cochrane Collaboration’s tool	VR therapy show immediate positive effects on functional locomotion in people with PD.
Lina C (2020) [21]	China	12 (360)	VR	AT	Gait speed Balance (BBS) Motor function (TUG) Activities of daily living (MBI)	Cochrane Collaboration’s tool	VR therapy may be valuable in improving the balance, gait speed, and ability to perform activities of daily living in patients with PD.
Chen Y (2020) [22]	China	14 (574)	VR	AT	Walking stability (DGI/FGA) Balance (BBS) Balance (TUG) Balance confidence (ABC)	PEDro scale	Compared with AT, VR therapy improved the balance (BBS). There was no significant difference on balance (TUG), balance confidence, and walking stability between the VR therapy groups and the AT groups.
Wang B (2019) [23]	China	12 (419)	VR	AT	Gait speed Stride/step length Walking distance (6MWT) Balance (BBS) Balance (TUG)	PEDro scale	This review demonstrated significant improvements in balance and stride length in PD patients who received VR compared with controls. There was no significant difference in gait speed and walking distance.
Santos P (2019) [24]	Brasil	5 (152)	VR	AT	Balance (BBS) Quality of life (PDQ-39)	PEDro scale	Combination VR and conventional physiotherapy was more effective on balance rehabilitation and quality of life of patients with PD.
Lei C (2019) [25]	China	16 (555)	VR	AT	Gait speed Stride/step length Walking stability (DGI) Balance (BBS) Balance (TUG) Balance confidence (ABC/FES) Motor function (UPDRS-III) Activities of daily living (UPDRS-II) Quality of life (PDQ-39/WHOQOL-OLD) Neuropsychiatric symptoms (BAI/BDI/HAMD) Cognitive function (DSF/MoCA)	Cochrane Collaboration’s tool	VR performed better than AT in step/stride length, balance, balance confidence, quality of life, and neuropsychiatric symptoms. There was no effect on the gait speed, DGI, motor function, cognitive function, and activities of daily living.
Dockx K (2016) [26]	Belgium	8 (263)	VR	AT; PT	Gait speed Stride/step length Balance (BBS) Balance (BBS/TUG/SLS) Quality of life (PDQ-39)	Cochrane Collaboration’s tool	In comparison to AT, VR may lead to a moderate improvement in step and stride length. VR and physiotherapy may have similar effects on gait, balance, and quality of life. In comparison to PT, VR therapy elicited greater improvements in gait, balance, and quality of life.
Harris DM (2015) [27]	Japan	2 (74)	VR	AT; PT.	Balance (BBS) Postural control (SOT)	PEDro scale	With the current available studies, the efficacy of VR therapy cannot be sufficiently determined for people with PD.

*VR* virtual reality, *AT* active intervention, *PT* passive intervention, *PD* Parkinson Disease, *PEDro* Physiotherapy Evidence Database, *DGI* Dynamic Gait Index, *FGA* Functional Gait Assessment, *6MWT* 6-Minute Walking Test, *BBS* Berg Balance Scale, *TUG* Timed Up and Go test, *ABC* Activities-Specific Balance Confidence scale, *FES* Falls Efficacy Scale, *FES-I* FES-international, *UPDRS-III* Unified Parkinson Disease Rating Scale part III, *PDQ-39* 39-item Parkinson Disease Questionnaire, *MBI* modified Barthel index, *HADS* Hospital Anxiety and Depression Scale, *HAMD* Hamilton Depression scale, *GDS-15* 15-item Geriatric Depression Scale, *BDI* Beck Depression Inventory, *WHOQOL-OLD* World Health Organization Quality of Life-Old, *BAI* Beck Anxiety Inventory, *DSF* Digit Span forward, *MoCA* Montreal Cognitive Assessment, *SLS* Single-Leg Stance Test, *SOT* sensory organization test

### Methodological quality of SR-MAs

Detailed information on the methodological quality of included SR-MAs was provided in Table 2. AMSTAR-2 score showed that one [25] (8.3%) review was of moderate quality, three [22, 23, 26] (25.0%) were low, and that of all the others [16–21, 24, 27] (66.7%) were critically low. The key factors affecting the quality of the literature included item 2 (only five reviews [16–18, 25, 26] had registered and had a protocol before performing the review), item 4 (seven reviews [16, 17, 19, 22, 23, 26, 27] used a comprehensive literature search strategy with searching references of relevant reviews or searching relevant gray literature), item 7 (two reviews [25, 26] provided a list of excluded studies and justified the exclusions), item 9 (all reviews [16–27] reported risk of bias use a satisfactory technique), item 11 (10 reviews [16, 18, 19, 21–27] conducted a statistical combination of results using appropriate methods), item 13 (all reviews [16–27] accounted for the risk of bias in the primary studies when interpreting the results of the reviews), and item 15 (three reviews [22, 23, 25] carried out an adequate investigation of publication bias study and discuss its impact on the review).

**Table 2:** Result of the AMSTAR-2 assessments

Study	AMSTAR-2																Quality
	Q1	Q2	Q3	Q4	Q5	Q6	Q7	Q8	Q9	Q10	Q11	Q12	Q13	Q14	Q15	Q16	
Sarasso E (2021) [16]	Y	Y	N	Y	Y	Y	N	Y	Y	N	Y	N	Y	Y	N	N	CL
Li R (2021) [17]	Y	Y	N	Y	Y	Y	N	Y	Y	N	N	N	Y	Y	N	Y	CL
Elena P (2021) [18]	Y	Y	N	PY	Y	Y	N	Y	Y	N	Y	N	Y	N	N	N	CL
Triegaardt J (2020) [19]	Y	N	Y	Y	N	N	N	PY	Y	N	Y	Y	Y	Y	N	Y	CL
Marotta N (2020) [20]	Y	N	N	PY	Y	Y	N	PY	Y	N	N	N	Y	N	N	Y	CL
Lina C (2020) [21]	Y	N	Y	PY	Y	Y	N	PY	Y	N	Y	Y	Y	Y	N	N	CL
Chen Y (2020) [22]	Y	N	Y	Y	Y	Y	PY	Y	Y	N	Y	Y	Y	Y	Y	N	L
Wang B (2019) [23]	Y	N	Y	Y	Y	Y	PY	Y	Y	N	Y	N	Y	Y	Y	Y	L
Santos P (2019) [24]	Y	N	Y	PY	Y	Y	N	Y	Y	N	Y	N	Y	Y	N	N	CL
Lei C (2019) [25]	Y	Y	Y	PY	Y	Y	Y	Y	Y	N	Y	N	Y	Y	Y	Y	M
Dockx K (2016) [26]	Y	Y	Y	Y	Y	Y	Y	Y	Y	Y	Y	Y	Y	Y	N	Y	L
Harris DM (2015) [27]	Y	N	Y	Y	Y	N	N	Y	Y	N	Y	Y	Y	N	N	Y	CL

*Y* yes, *PY* partial yes, *N* no, *CL* critically low, *L* low, *M* moderate, *H* high

Q1: Did the research questions and inclusion criteria for the review include the components of PICO?

Q2: Did the report of the review contain an explicit statement that the review methods were established prior to the conduct of the review and did the report justify any significant deviations from the protocol?

Q3: Did the review authors explain their selection of the study designs for inclusion in the review?

Q4: Did the review authors use a comprehensive literature search strategy?

Q5: Did the review authors perform study selection in duplicate?

Q6: Did the review authors perform data extraction in duplicate?

Q7: Did the review authors provide a list of excluded studies and justify the exclusions?

Q8: Did the review authors describe the included studies in adequate detail?

Q9: Did the review authors use a satisfactory technique for assessing the risk of bias (RoB) in individual studies that were included in the review?

Q10: Did the review authors report on the sources of funding for the studies included in the review?

Q11: If meta-analysis was performed, did the review authors use appropriate methods for statistical combination of results?

Q12: If meta-analysis was performed, did the review authors assess the potential impact of RoB in individual studies on the results of the meta-analysis or other evidence synthesis?

Q13: Did the review authors account for RoB in individual studies when interpreting/discussing the results of the review?

Q14: Did the review authors provide a satisfactory explanation for, and discussion of, any heterogeneity observed in the results of the review?

Q15: If they performed quantitative synthesis, did the review authors carry out an adequate investigation of publication bias (small study bias) and discuss its likely impact on the results of the review?

Q16: Did the review authors report any potential sources of conflict of interest, including any funding they received for conducting the review?

*Critical domains*: Q2, Q4, Q7, Q9, Q11, Q13, and Q15. *High*: No or one non-critical weakness. *Moderate*: More than one non-critical weakness. *Low*: One critical flaw with or without non-critical weaknesses. *Critically low*: More than one critical flaw with or without non-critical weaknesses

### Effect of interventions

We found marked heterogeneity of the evaluated comparisons and measured outcomes among the included reviews. Various comparison modes in included reviews and key findings are summarized below.

### Comparison 1: VR therapy versus AT

**Fig. 2:**
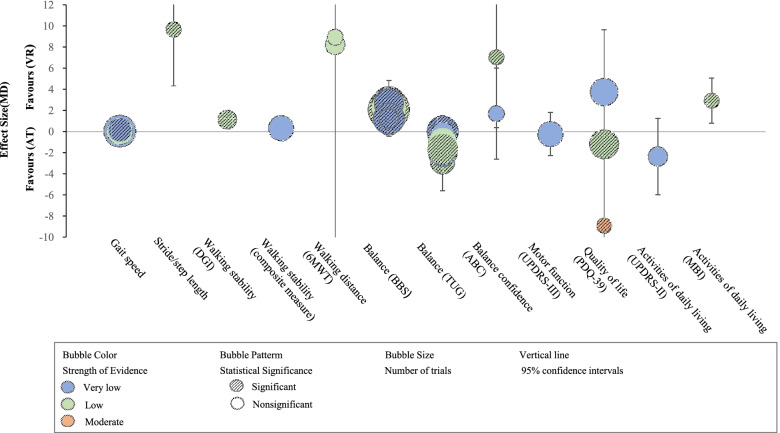
Evidence map of effectiveness (MD) of virtual reality therapy for patients with Parkinson’s disease compared with active intervention. Note. MD, mean difference; AT, active intervention; VR, virtual reality; DGI, Dynamic Gait Index; 6MWT, Six-Minute Walk Test; BBS, Berg Balance Scale; TUG, Timed Up and Go test; ABC, Activities-specific Balance Confidence scale; UPDRS, Unified Parkinson Disease Rating Scale; PDQ-39, 39-Item Parkinson’s Disease Questionnaire; MBI, modified Barthel index

**Fig. 3:**
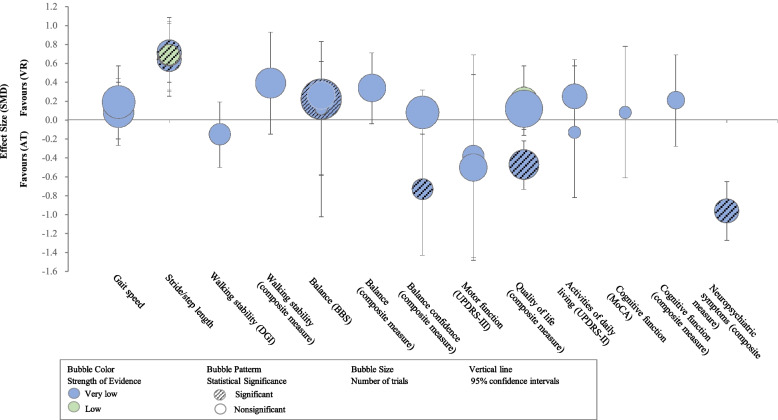
Evidence map of effectiveness (SMD) of virtual reality therapy for patients with Parkinson’s disease compared with active intervention. Note. SMD, standard mean difference; AT, active intervention; VR, virtual reality; DGI, Dynamic Gait Index; BBS, Berg Balance Scale; UPDRS, Unified Parkinson Disease Rating Scale; MoCA, Montreal Cognitive Assessment

An overview of the review result summary is provided in Table 3. Figures 2 and 3 presented the evidence map of effectiveness for VR therapy compared to AT in the patients with PD.

**Table 3:** Summary of the effectiveness of virtual reality therapy compared to active intervention by outcomes in Parkinson’s disease

Outcomes	Study	Effect estimation (95% *CI*)	Studies (participants)	Certainty of the evidence (GRADE)
Gait speed	Sarasso E (2021) [16]	*MD* 0.03 (−0.01, 0.07)	8 (279)	⊕〇〇〇 Very low^a,c,f^
Gait speed	Triegaardt J (2020) [19]	*SMD* 0.08 (−0.27, 0.44)	6 (209)	⊕〇〇〇 Very low^a,c^
Gait speed	Lina C (2020) [21]	*MD* 0.13(0.02, 0.24)	4 (174)	⊕〇〇〇 Very low^a,c^
Gait speed	Wang B (2019) [23]	*MD* −0.00 (−0.06, 0.06)	5 (203)	⊕⊕〇〇 Low^c,d^
Gait speed	Lei C (2019) [25]	*SMD* 0.19 (−0.03, 0.40)	7 (347)	⊕〇〇〇 Very low^a,c,f^
Gait speed	Dockx K (2016) [26]	*SMD* 0.18 (−0.20, 0.57)	3 (106)	⊕⊕〇〇 Low^c,d^
Stride/step length	Sarasso E (2021) [16]	*SMD* 0.64 (0.25, 1.02)	4 (110)	⊕〇〇〇 Very low^a,c,f^
Stride/step length	Triegaardt J (2020) [19]	*SMD* 0.70 (0.32, 1.08)	4 (116)	⊕〇〇〇 Very low^a,c^
Stride/step length	Wang B (2019) [23]	*MD* 9.65 (4.31, 14.98)	2 (79)	⊕⊕〇〇 Low^a,c,g^
Stride/step length	Lei C (2019) [25]	*SMD* 0.72 (0.40, 1.04)	4 (166)	⊕〇〇〇 Very low^c,d,f^
Stride/step length	Dockx K (2016) [26]	*SMD* 0.69 (0.30, 1.08)	3 (106)	⊕⊕〇〇 Low^c,d^
Walking stability (DGI)	Elena P (2021) [18]	*MD* 1.13 (0.35, 1.92)	3 (176)	⊕⊕〇〇 Low^c,d^
Walking stability (DGI)	Lei C (2019) [25]	*SMD* −0.15 (−0.50, 0.19)	3 (130)	⊕〇〇〇 Very low^a,c^
Walking stability (DGI/FGA)	Sarasso E (2021) [16]	*SMD* 0.39 (−0.15, 0.93)	6 (207)	⊕〇〇〇 Very low^a,b,c.f^
Walking stability (DGI/FGA)	Chen Y (2020) [22]	*MD* 0.31 (−0.56, 1.19)	5 (220)	⊕〇〇〇 Very low^a,b,c,f^
Walking distance (6MWT)	Sarasso E (2021) [16]	*MD* 8.20 (−17.28, 33.69)	3 (72)	⊕⊕〇〇 Low^a,c,g^
Walking distance (6MWT)	Wang B (2019) [23]	*MD* 8.91 (−43.43, 61.13)	2 (45)	⊕⊕〇〇 Low^a,c,g^
Balance (BBS)	Sarasso E (2021) [16]	*MD* 2.09 (0.86, 3.33)	14 (430)	⊕⊕〇〇 Low ^a,b,g^
Balance (BBS)	Elena P (2021) [18]	*MD* 2.64 (0.45, 4.83)	7 (281)	⊕〇〇〇 Very low^a,b,c,g^
Balance (BBS)	Triegaardt J (2020) [19]	*SMD* 0.26 (−1.02, 0.62)	5 (166)	⊕〇〇〇 Very low^a,b,c^
Balance (BBS)	Lina C (2020) [21]	*MD* 2.28 (1.39, 3.16)	9 (281)	⊕⊕〇〇 Low^a,c,g^
Balance (BBS)	Chen Y (2020) [22]	*MD* 1.23 (0.15, 2.31)	8 (266)	⊕〇〇〇 Very low^a,b,c^
Balance (BBS)	Wang B (2019) [23]	*MD* 2.69 (1.37, 4.02)	9 (299)	⊕〇〇〇 Very low^a,b,c,g^
Balance (BBS)	Santos P (2019) [24]	*MD* 1.24 (0.24, 2.25)	3 (72)	⊕〇〇〇 Very low^a,c^
Balance (BBS)	Lei C (2019) [25]	*SMD* 0.22 (0.01, 0.42)	11 (360)	⊕〇〇〇 Very low^a,c^
Balance (BBS)	Dockx K (2016) [26]	*MD* 0.55 (−0.48, 1.58)	3 (86)	⊕⊕〇〇 Low^c,d^
Balance (BBS)	Harris DM (2015) [27]	*SMD* 0.12 (−0.58, 0.83)	1 (32)	⊕〇〇〇 Very low^a,c^
Balance (TUG)	Sarasso E (2021) [28]	*MD* −1.55 (−3.06, −0.04)	8 (236)	⊕〇〇〇 Very low^c,d,e^
Balance (TUG)	Elena P (2021) [18]	*MD* −0.98 (−2.21, 0.26)	6 (205)	⊕⊕〇〇 Low^c,d^
Balance (TUG)	Lina C (2020) [21]	*MD* −1.66 (−2.74, −0.58)	7 (190)	⊕⊕〇〇 Low^c,d^
Balance (TUG)	Chen Y (2020) [22]	*MD* −0.18 (−1.37, 1.00)	4 (120)	⊕〇〇〇 Very low^b,c,d^
Balance (TUG)	Wang B (2019) [23]	*MD* −2.86 (−5.60, −0.12)	5 (144)	⊕⊕〇〇 Low^b,c,d,g^
Balance (TUG)	Lei C (2019) [25]	*MD* −1.95 (−2.81, −1.08)	7 (237)	⊕〇〇〇 Very low^b,c,d^
Balance (BBS/TUG/SLS)	Dockx K (2016) [26]	*SMD* 0.34 (−0.04, 0.71)	5 (155)	⊕〇〇〇 Very low^c,d,f^
Balance confidence (ABC)	Elena P (2021) [18]	*MD* 7.03 (0.36, 13.69)	2 (115)	⊕⊕〇〇 Low^a,c,g^
Balance confidence (ABC)	Chen Y (2020) [22]	*MD* 1.69 (−2.62, 6.01)	2 (115)	⊕〇〇〇 Very low^a,c^
Balance confidence (ABC/FES/FES-I)	Sarasso E (2021) [28]	*SMD* 0.08 (−0.15, 0.32)	7 (334)	⊕〇〇〇 Very low^a,c,f^
Balance confidence (ABC/FES)	Lei C (2019) [25]	*SMD* −0.73 (−1.43, −0.02)	3 (104)	⊕〇〇〇 Very low^b,c,d,f^
Motor function (UPDRS-III)	Sarasso E (2021) [28]	*MD* −0.25 (−2.28, 1.79)	5 (164)	⊕〇〇〇 Very low^b,c,d^
Motor function (UPDRS-III)	Triegaardt J (2020) [19]	*SMD* −0.38 (−1.45, 0.69)	3 (75)	⊕〇〇〇 Very low^b,c,d^
Motor function (UPDRS-III)	Lei C (2019) [25]	*SMD* −0.50 (−1.48, 0.48)	5 (164)	⊕〇〇〇 Very low^a,b,c^
Quality of life (PDQ-39)	Elena P (2021) [18]	*MD* −1.21 (−1.68, −0.73)	7 (207)	⊕〇〇 Low^c,d^
Quality of life (PDQ-39)	Santos P (2019) [24]	*MD* −8.90 (−15.22, −2.58)	2 (56)	⊕⊕⊕〇 Moderate^c,d,g^
Quality of life (PDQ-39)	Dockx K (2016) [26]	*MD* 3.73 (−2.16, 9.61)	6 (106)	⊕〇〇〇 Very low^a,b,c,g^
Quality of life (PDQ-39/PDQ-8)	Sarasso E (2021) [28]	*SMD* 0.12 (−0.10, 0.35)	9 (303)	⊕〇〇〇 Very low^a,c,f^
Quality of life (PDQ-39/PDQ-8)	Triegaardt J (2020) [19]	*SMD* 0.20 (−0.16, 0.57)	5 (176)	⊕〇〇 Low^c,d^
Quality of life (PDQ-39/WHOQOL-OLD)	Lei C (2019) [25]	*SMD* −0.47 (−0.73, −0.22)	6 (248)	⊕〇〇〇 Very low^c,d,f^
Activities of daily living (UPDRS-II)	Elena P (2021) [18]	*MD* −2.37 (−5.97, 1.23)	3 (101)	⊕〇〇〇 Very low^a,b,c,g^
Activities of daily living (UPDRS-II)	Triegaardt J (2020) [19]	*SMD* −0.13 (−0.82, 0.57)	1 (32)	⊕〇〇〇 Very low^a,c^
Activities of daily living (UPDRS-II)	Lei C (2019) [25]	*SMD* 0.25 (−0.14, 0.64)	4 (103)	⊕〇〇〇 Very low^a,c^
Activities of daily living (MBI)	Lina C (2020) [21]	*MD* 2.93 (0.80, 5.06)	2 (51)	⊕⊕〇〇 Low^a,c,g^
Cognitive function (MoCA)	Triegaardt J (2020) [19]	*SMD* 0.08 (−0.61, 0.78)	1 (32)	⊕〇〇〇 Very low^a,c^
Cognitive function (DSF/MoCA)	Lei C (2019) [25]	*SMD* 0.21 (−0.28, 0.69)	2 (68)	⊕〇〇〇 Very low^a,c,f^
Neuropsychiatric symptoms (BAI/BDI/HAMD)	Lei C (2019) [25]	*SMD* −0.96 (−1.27, −0.65)	4 (184)	⊕〇〇〇 Very low^c,d,f^

*CI* confidence intervals, *GRADE* Grading of Recommendations Assessment, Development and Evaluation, *MD* mean difference, *SMD* standard mean difference, *DGI* Dynamic Gait Index, *FGA* Functional Gait Assessment, *6-WMT* 6-Minute Walking Test, *BBS* Berg balance scale, *TUG* Timed Up and Go test, *SLS* Single-Leg Stance Test, *ABC* Activities-Specific Balance Confidence scale, *FES* Falls Efficacy Scale, *FES-I* FES-international, *UPDRS-III* Unified Parkinson Disease Rating Scale part III, *PDQ-39* 39-item Parkinson Disease Questionnaire, *WHOQOL-OLD* World Health Organization Quality of Life-Old, *MBI* modified Barthel index, *MoCA* Montreal Cognitive Assessment, *DSF* Digit Span forward, *BAI* Beck Anxiety Inventory, *BDI* Beck Depression Inventory, *HAMD* Hamilton Depression scale

GRADE Working Group grades of evidence—high certainty: we are very confident that the true effect lies close to that of the estimate of the effect. Moderate certainty: we are moderately confident in the effect estimate: the true effect is likely to be close to the estimate of the effect, but there is a possibility that it is substantially different. Low certainty: our confidence in the effect estimate is limited: the true effect may be substantially different from the estimate of the effect. Very low certainty: we have very little confidence in the effect estimate: the true effect is likely to be substantially different from the estimate of effect

^a^High risk of bias in at least a half of studies included within the analysis, hence bias is highly likely. Therefore, the certainty of evidence was downgraded by two levels due to the methodological limitations (risk of bias)

^b^Substantial heterogeneity among trials (*I*^2^ equal or more than 50%, equal or less than 90%). Therefore, the certainty of evidence was downgraded by one level (inconsistency)

^c^The total population size was small (<400). Therefore, the certainty of evidence was downgraded by one level (imprecision)

^d^High risk of bias in less than a half of studies included within the analysis, hence bias is highly likely. Therefore, the certainty of evidence was downgraded by one level due to the methodological limitations (risk of bias)

^e^Considerable heterogeneity among trials (*I*^2^>90%). Therefore, the certainty of evidence was downgraded by two levels (inconsistency)

^f^Different ways of assessment were used across studies. Therefore, the certainty of evidence was downgraded by one level (indirectness)

^g^The estimated effect was large reaching a plausible clinically relevant magnitude. Therefore, the certainty of evidence was upgraded by one level (other consideration, large effect)

Five reviews [16, 19, 23, 25, 26] reported the stride/step length and concluded that VR therapy had a greater improvement of stride/step length compared with AT. The balance function was assessed by Berg Balance Scale (BBS) and Timed Up and Go test (TUG) in ten [16, 18, 19, 21–27] and six [16, 18, 21–23, 25] reviews, respectively, and the majority (7/10, 4/6) indicated a significant difference between VR therapy and AT, whereby VR therapy was shown to be superior. Only one review [25] investigated the effect of VR therapy on neuropsychiatric symptoms and found a significant improvement (*SMD* = −0.96, 95% CI = −1.27 to −0.65, very low-certainty evidence) compared with AT. The low to very low certainty of evidence across reviews means it was not possible to state whether more benefit of VR therapy on stride/step length, balance function, and neuropsychiatric symptoms when compared to AT.

The results regarding gait speed, walking stability, balance confidence, quality of life, and activities of daily living were mixed and provided no convincing evidence of the effect of VR therapy versus AT on these areas.

We found no significant difference between VR and AT on walking distance, motor function, and cognitive function. Most reviews described similar improvements in both exercise groups.

### Comparison 2: VR therapy versus PT

An overview of the review result summary is provided in Table 4.

**Table 4:** Summary of the effectiveness of virtual reality therapy compared to passive intervention by outcomes in Parkinson’s disease

Outcomes	Study	Effect estimation (95 % CI)	Studies (participants)	Certainty of the evidence (GRADE)
Gait speed	Triegaardt J (2020) [19]	*SMD* 1.43 (0.51, 2.34)	1 (24)	⊕⊕〇〇 Low^c,d^
Stride/step length	Triegaardt J (2020) [19]	*SMD* 1.27 (0.38, 2.16)	1 (24)	⊕⊕〇〇 Low^c,d^
Balance (BBS)	Triegaardt J (2020) [19]	*SMD* 1.02 (0.38, 1.65)	2 (44)	⊕⊕〇〇 Low^a,c,g^
Balance (BBS/TUG)	Dockx K (2016) [26]	*SMD* 1.02 (0.38, 1.65)	2 (44)	⊕〇〇〇 Very low^a,c^
Activities of daily living (MBI)	Triegaardt J (2020) [19]	*SMD* 0.96 (0.02, 1.89)	1 (20)	⊕〇〇〇 Very low^a,c^
Postural control (SOT)	Harris DM (2015) [27]	*SMD* 2.57(1.53, 3.60)	1 (28)	⊕⊕⊕〇 Moderate^c,d,g^

*CI* confidence intervals, *GRADE* Grading of Recommendations Assessment, Development and Evaluation, *SMD* standard mean difference, *BBS* Berg balance scale, *TUG* Timed Up and Go test, *MBI* modified Barthel index, *SOT* sensory organization test

GRADE Working Group grades of evidence—high certainty: we are very confident that the true effect lies close to that of the estimate of the effect. Moderate certainty: we are moderately confident in the effect estimate: the true effect is likely to be close to the estimate of the effect, but there is a possibility that it is substantially different. Low certainty: our confidence in the effect estimate is limited: the true effect may be substantially different from the estimate of the effect. Very low certainty: we have very little confidence in the effect estimate: the true effect is likely to be substantially different from the estimate of effect

^a^High risk of bias in at least a half of studies included within the analysis, hence bias is highly likely. Therefore, the certainty of evidence was downgraded by two levels due to the methodological limitations (risk of bias)

^b^Substantial heterogeneity among trials (*I*^2^ equal or more than 50%, equal or less than 90%). Therefore, the certainty of evidence was downgraded by one level (inconsistency)

^c^The total population size was small (<400). Therefore, the certainty of evidence was downgraded by one level (imprecision)

^d^High risk of bias in less than a half of studies included within the analysis, hence bias is highly likely. Therefore, the certainty of evidence was downgraded by one level due to the methodological limitations (risk of bias)

^e^Considerable heterogeneity among trials (*I*^2^>90%). Therefore, the certainty of evidence was downgraded by two levels (inconsistency)

^f^Different ways of assessment were used across studies. Therefore, the certainty of evidence was downgraded by one level (indirectness)

^g^The estimated effect was large reaching a plausible clinically relevant magnitude. Therefore, the certainty of evidence was upgraded by one level (other consideration, large effect)

We found three reviews investigating VR therapy versus PT in participants with PD. Triegaardt et al. [19] reported that VR therapy had greater effects on gait speed, stride/step length, balance function, and activities of daily living compared with PT. Dockx et al. [26] showed a significant benefit of VR exercise on balance as a composite measure (*SMD* 1.02, 95% *CI* 0.38 to 1.65) compared to PT. The evidence [27] derived from a single study showed an improvement in postural control (*SMD* 2.57, 95% *CI* 1.53 to 3.60) after VR therapy. Given the moderate to very low certainty of the evidence and limited data, we were unable to make any conclusion on the effect of VR therapy versus PT on function in people with PD.

### Comparison 3: VR therapy versus controls (mixed AT and PT)

One review [17] revealed that training significantly improved balance (*g* = 0.66, *P* < 0.001), quality of life (*g* = 0.28, *P* = 0.015), activities of daily living (*g* = 0.62, *P* < 0.001), and neuropsychiatric symptoms (*g* = 0.67, *P* = 0.021) compared to the control group. A second review [20] reported that Kinect and Wii showed immediate positive effects on functional locomotion in people with PD. However, we considered this pooled comparison to be flawed as the combination of AT/PT groups was in our view problematic given the likely differences in underlying effect sizes for these two groups in head-to-head comparisons with VR therapy. We therefore have not presented this result in table. Both reviews reporting pooled analysis rated the quality of the evidence as low to very low.

## Discussion

### Summary of main findings

Based on the current findings, VR therapy induced (1) increased benefits on stride/step length, balance, and neuropsychiatric symptoms as compared with AT and (2) greater effects on gait speed, stride/step length, balance, activities of daily living, and postural control as compared with PT in people with PD.

Three reviews [16, 23, 26] formally rated the evidence using the GRADE approach and self-rated the evidence as very low quality. The remaining reviews [17–22, 24, 25, 27] did not explicitly use the GRADE approach; however, following consideration of factors such as their risk of bias appraisal results and the size of included studies, we rated them also as offering very low certainty of evidence. In addition, the overall quality of methodology of included reviews was also unsatisfactory.

We found that despite included reviews spanning decades of research, this overview was unable to offer any reliable estimate of the effect of VR therapy in terms of gait, balance, motor function, quality of life, activities of daily living, cognitive function, neuropsychiatric symptoms, and postural control.

In addition, we investigated potential causes of inconsistent results for outcome as follows: (1) Participants’ characteristics and clinical stages (Hoehn-Yahr, H&Y) may be different. Sarasso et al. [16] found the larger effect of VR-based balance training was observed in patients with greater balance deficits and disease severity (H&Y > 2) at baseline. Patients with greater balance deficits are usually in a more advanced phase of the disease, having also initial executive-attentive and visuospatial dysfunctions that could influence balance. In these patients, VR might have the potential to train both motor and cognitive domains (particularly executive-attentive and visuospatial functions) leading to a greater balance improvement. (2) Different VR modalities may be a key factor. Sarasso et al. [16] reported that VR rehabilitation-specific systems, designed and customized for a rehabilitative goal, are more effective than non-specific systems, such as commercial exergames, to improve balance in PD patients. This finding is supported by similar preliminary evidence in stroke patients [28] and gives reason for a continuous development and implementation of customizable VR systems. (3) There was high heterogeneity in outcome measures, making it difficult to make valid comparisons between different reviews. For example, activities of daily living assessed with Unified Parkinson Disease Rating Scale part II (UPDRS-II) [18, 19, 25] or modified Barthel index (MBI) [21] did not yield consistent results even under the same comparison mode.

### Strengths and limitation of the overview

To the best of our knowledge, our study is the first overview of SR-MAs to explore the effect of VR therapy on PD rehabilitation, which may have certain reference value for the clinical practice. In addition, the findings of this overview were based on relatively recent evidence, as all studies were published in the last 6 years. Moreover, this overview included SR-MAs of RCTs using strict inclusion standards in order to reduce the risk of bias. However, this study has several limitations. First, the methodological quality and evidence quality of the included SR-MAs were generally very low; thus, results based on primary studies should be interpreted with caution. Second, we only searched English databases, so SR-MAs published in other languages that met the inclusion criteria may have been missed. Third, there was a great heterogeneity of outcomes across the included reviews, which limited the ability to interpret overall pooled estimates. For future research, it would be necessary at least to define a homogenous outcome core set to assess the effect of VR therapy in PD patients. Fourth, the combined effects of VR therapy with any type of ATs should be compared with the same type of AT so that the additional benefits of VR therapy can be elucidated. Unfortunately, the meta-analyses often pooled trials with highly heterogeneous interventions (i.e., VR therapy/VR therapy combined with other ATs), which makes interpretation of their results very difficult. However, our overview cannot avoid this limitation and our findings must be interpreted with caution. Fifth, our overall GRADE assessment was based on a combination of assessments made by the systematic review authors and ourselves. This combination may entail inconsistency in assessments, as reliability between the assessment made by the authors of the systematic reviews and our research group is unknown. Therefore, our overview cannot avoid this limitation and our findings must be interpreted with caution.

## Conclusion

We found the methodological quality of the reviews and the certainty of the evidence within them was poor. We were therefore unable to conclude with any confidence that, in people with PD, VR therapy is beneficial for gait, balance function, balance confidence, motor function, quality of life, activities of daily living, cognitive function, neuropsychiatric symptoms, and postural control. Rigorous-designed, high-quality RCTs with larger sample sizes are needed to further verify the effectiveness of VR therapy in the treatment of PD.

## Supplementary Information

**Additional file 1.** PRISMA 2009 Checklist.

**Additional file 2.** Search Strategy for PubMed.

## Data Availability

All data generated or analyzed during this study are included in this published article [and its supplementary information files].

## Abbreviations

AMSTAR-2: Assessment of Multiple Systematic Reviews 2
AT: Active intervention
GRADE: Grading of Recommendations, Assessment, Development and Evaluation
MAs: Meta-analyses
PD: Parkinson disease
PRISMA: Preferred Reporting Items for Systematic Reviews and Meta-Analyses
PT: Passive interventions
RCT: Randomized controlled trials
SRs: Systematic reviews
VR: Virtual reality
