# Supplementary material for: The effectiveness of virtual reality for rehabilitation of Parkinson disease: an overview of systematic reviews with meta-analyses
Source: Syst Rev. 2022 Mar 19;11:50. doi: 10.1186/s13643-022-01924-5 (PMC8934460; doi:10.1186/s13643-022-01924-5)
Supplement: Supplementary file 2 — Additional file 2. Search Strategy for PubMed. [file 13643_2022_1924_MOESM2_ESM.docx]

**Additional file 2.**

**Search strategy: take the search process via Pubmed as an example**

| **Query** | **Search term** |
| --- | --- |
| **#1** | **"Parkinson Disease"[Mesh]** |
| **#2** | **(Parkinson Disease[Title/Abstract]) OR (Parkinson's Disease[Title/Abstract]) OR (Parkinsonism[Title/Abstract]) OR (Paralysis Agitans[Title/Abstract]) OR (Shaking Palsy[Title/Abstract])** |
| **#3** | **#1 OR #2** |
| **#4** | **"Virtual Reality"[Mesh] OR "Virtual Reality Exposure Therapy"[Mesh] OR "Exergaming"[Mesh]** |
| **#5** | **(VR[Title/Abstract]) OR (Virtual[Title/Abstract]) OR (Virtuality[Title/Abstract]) OR (Augmented[Title/Abstract]) OR (Computer*[Title/Abstract]) OR (Software[Title/Abstract]) OR (Gaming[Title/Abstract]) OR (Game[Title/Abstract]) OR (User‐computer interface[Title/Abstract]) OR (Simulation[Title/Abstract]) OR (Exergam*[Title/Abstract]) OR (Reality system[Title/Abstract]) OR (Interactive[Title/Abstract]) OR (X-box[Title/Abstract]) OR (Kinect[Title/Abstract]) OR (Nintendo[Title/Abstract]) OR (Wii[Title/Abstract])** |
| **#6** | **#4 OR #5** |
| **#7** | **"Meta-Analysis as Topic"[Mesh] OR (Meta-Analysis [Publication Type])** |
| **#8** | **(meta analysis[Title/Abstract]) OR (meta analyses[Title/Abstract]) OR (metaanalysis[Title/Abstract]) OR (metaanalyses[Title/Abstract]) OR (metanalysis[Title/Abstract]) OR (metanalyses[Title/Abstract]) OR (met-analysis[Title/Abstract]) OR (met-analyses[Title/Abstract]) OR (meta-study[Title/Abstract]) OR (meta-studies[Title/Abstract]) OR (meta study[Title/Abstract]) OR (meta studies[Title/Abstract]) OR (data pooling[Title/Abstract]) OR (data poolings[Title/Abstract]) OR (clinical trial overview[Title/Abstract]) OR (clinical trial overviews[Title/Abstract])** |
| **#9** | **"Systematic Reviews as Topic"[Mesh] OR (Systematic Review [Publication Type])** |
| **#10** | **(systematic review [Title/Abstract]) OR (systematic reviews[Title/Abstract]) OR (systematic study[Title/Abstract]) OR (systematic studies [Title/Abstract])** |
| **#11** | **#7 OR #8 OR #9 OR #10** |
| **#12** | **#3 AND #6 AND #11** |
